# White light emission in 0D halide perovskite [(CH_3_)_3_S]_2_SnCl_6_·H_2_O crystals through variation of doping ns^2^ ions

**DOI:** 10.1007/s12200-024-00109-3

**Published:** 2024-02-20

**Authors:** Yitong Lin, Yu Zhong, Yangpeng Lin, Jiawei Lin, Lei Pang, Zhilong Zhang, Yi Zhao, Xiao-Ying Huang, Ke-Zhao Du

**Affiliations:** 1 Fujian Provincial Key Laboratory of Advanced Materials Oriented Chemical Engineering, Collage of Chemistry and Material Science, Fujian Normal University, Fuzhou, 350007 China; 2 State Key Laboratory of Structural Chemistry, Fujian Institute of Research on the Structure of Matter, Chinese Academy of Sciences, Fuzhou, 350002 China; 3 Qinghai Environmental Monitoring Center, Xining, 810000 China; 4 Strait Institute of Flexible Electronics (SIFE, Future Technologies), Fujian Normal University and Strait Laboratory of Flexible Electronics (SLoFE), Fuzhou, 350007 China; 5 Wuhan National Laboratory for Optoelectronics, Huazhong University of Science and Technology, Wuhan, 430074 China

**Keywords:** 0D, Metal halide, White light, Perovskite, Ions doping, Excitation dependent

## Abstract

With the rapid development of white LEDs, the research of new and efficient white light emitting materials has attracted increasing attention. Zero dimensional (0D) organic–inorganic hybrid metal halide perovskites with superior luminescent property are promising candidates for LED application, due to their abundant and tailorable structure. Herein, [(CH_3_)_3_S]_2_SnCl_6_·H_2_O is synthesized as a host for dopant ions Bi^3+^ and Sb^3+^. The Sb^3+^ doped, or Bi^3+^/Sb^3+^ co-doped, [(CH_3_)_3_S]_2_SnCl_6_·H_2_O has a tunable optical emission spectrum by means of varying dopant ratio and excitation wavelength. As a result, we can achieve single-phase materials suitable for emission ranging from cold white light to warm white light. The intrinsic mechanism is examined in this work, to clarify the dopant effect on the optical properties. The high stability of title crystalline material, against water, oxygen and heat, makes it promising for further application.

**Graphical Abstract:**

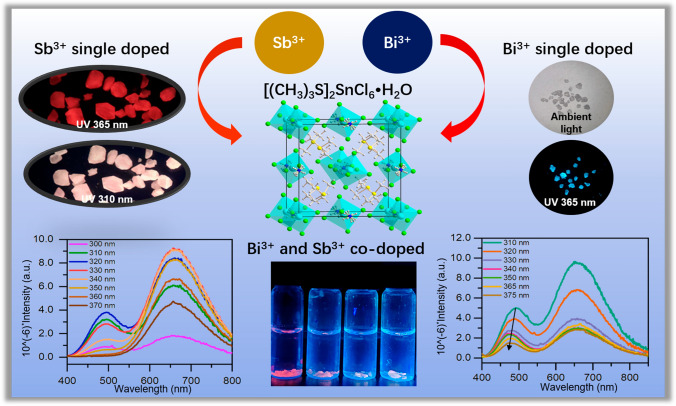

**Supplementary Information:**

The online version contains supplementary material available at 10.1007/s12200-024-00109-3.

## Introduction

Recently, solid-state lighting technology has experienced rapid development, in fields such as inorganic light emitting diodes (LEDs), organic light emitting diodes (OLEDs), polymer light emitting diodes (PLEDs) [1–3]. LEDs are more efficient and energy-saving than traditional lighting source [4] so that white LED has already largely replaced traditional lighting equipment (including incandescent lamps and fluorescent lamps). According to the US Department of Energy’s Solid-State Lighting report, electricity consumption for lighting is forecast to decrease by 25% between 2016 and 2035 [5].

The excellent optoelectronic properties of metal halide perovskites (MHPs) make them have potential applications in solar cells [6–8] and LEDs [9–11]. MHPs have different structural dimensionality ranging from zero-dimensional (0D) to three-dimensional (3D) [12–17]. The 0D metal halide has a soft lattice and a large Huang-Rhys factor (S), which is conducive to electron–phonon coupling [18, 19]. Thus, 0D metal halides have a stronger ability to form self-trapped excitons (STEs) than 3D ones do. The STEs have the potential to result in a broad emission with a large Stokes shift, with negligible self-absorption [18]. A_2_NX_6_ (A is a monovalent organic/inorganic cation, N is a tetravalent metal ion, and X is a halide ion) is a typical kind of 0D vacancy ordered MHP, exhibiting fruitful structures and good tolerance for guest ions [20–22]. For example, Cs_2_SnCl_6_ with excellent high stability has attracted considerable research attention. Various guest ions can be doped into Cs_2_SnCl_6_ leading to a distinctive performance. Bi^3+^, Te^4+^, and Sb^3+^ guest ions doped in Cs_2_SnCl_6_ show blue, yellow and red–orange light emission, respectively [23–28]. Through color addition, white light emission can be obtained by co-doping of Bi^3+^ and Te^4+^ ions [28, 29]. Based on the blue emission from the defect of Cs_2_SnCl_6_ structure, Ce^3+^ doped into Cs_2_SnCl_6_ induces an enhanced blue emission [30], while La^3+^ doped into Cs_2_SnCl_6_ achieves complementary white light [31]. Similar luminescence tuning by guest ions has also been reported in (NH_4_)_2_SnCl_6_ [32, 33].

In addition to the inorganic cation in A_2_SnX_6_ systems, organic cations have the potential to generate rich crystal structures due to the tailorable structure of organic cations. For example, (C_6_N_2_H_16_Cl)_2_SnCl_6_ (C_6_N_2_H_16_Cl = 2,6-dimethylpiperazine chloride) exhibits blue emission from STEs [34], while (4-APEA)_2_SnBr_6_ (4-APEA = 2-(4-aminophenyl) ethylammonium) provides yellow emission [35]. Sb^3+^ doped (C_10_H_16_N_2_)SnCl_6_ (C_10_H_16_N_2_ = 1-phenylpiperazine) induces ultra-broadband emission (400–900 nm) with 77% photoluminescence quantum yield (PLQY) [36]. However, most of the organic cation in A_2_SnX_6_ is protonated. The aprotic cation study is still in its infancy. To maintain the 216-type highly symmetric perovskite structure, in this work we introduce [(CH_3_)_3_S]^+^ into the A_2_SnX_6_ crystal structure. Compared with protonated organic cations, sulfonium cations have several advantages including [37]: (1) the characteristic of proton inertness, so that they do not undergo dehydrogenation reactions initiated by unstable free radicals or bases; (2) the large atomic size of S results in it combining closely with inorganic halide anions, resulting in an improved stability; (3) the use of aprotic sulfonium cation increases the moisture resistance of the perovskite structure. The study of diverse organic molecular structures can help to identify an organic cation that balances device performance and stability.

In this work, the organic–inorganic hybrid tin-based perovskite SSC (SSC = [(CH_3_)_3_S]_2_SnCl_6_·H_2_O) was synthesized. Bi^3+^ and Sb^3+^ ions were doped into SSC to tune the photoluminescence (PL), resulting in single-phase white light crystals Sb^3+^ doped [(CH_3_)_3_S]_2_SnCl_6_·H_2_O (Sb^3+^@SSC) and Bi^3+^/Sb^3+^ co-doped [(CH_3_)_3_S]_2_SnCl_6_·H_2_O (Bi^3+^/Sb^3+^@SSC). Their emission spectra are excitation-dependent. Thus, a series of high-quality white light emitting crystals with controllable color temperature can be obtained by adjusting the excitation wavelength. Compared with Sb^3+^@SSC, an enhanced blue light component, and a longer wavelength excitation (384 nm) for white light emission, can be achieved from Bi^3+^ in Bi^3+^/Sb^3+^@SSC. The title crystals have good acid resistance, water resistance and oxygen resistance, providing application potential for white light emitting diodes (WLEDs).

## Results and discussion

**Fig. 1:**
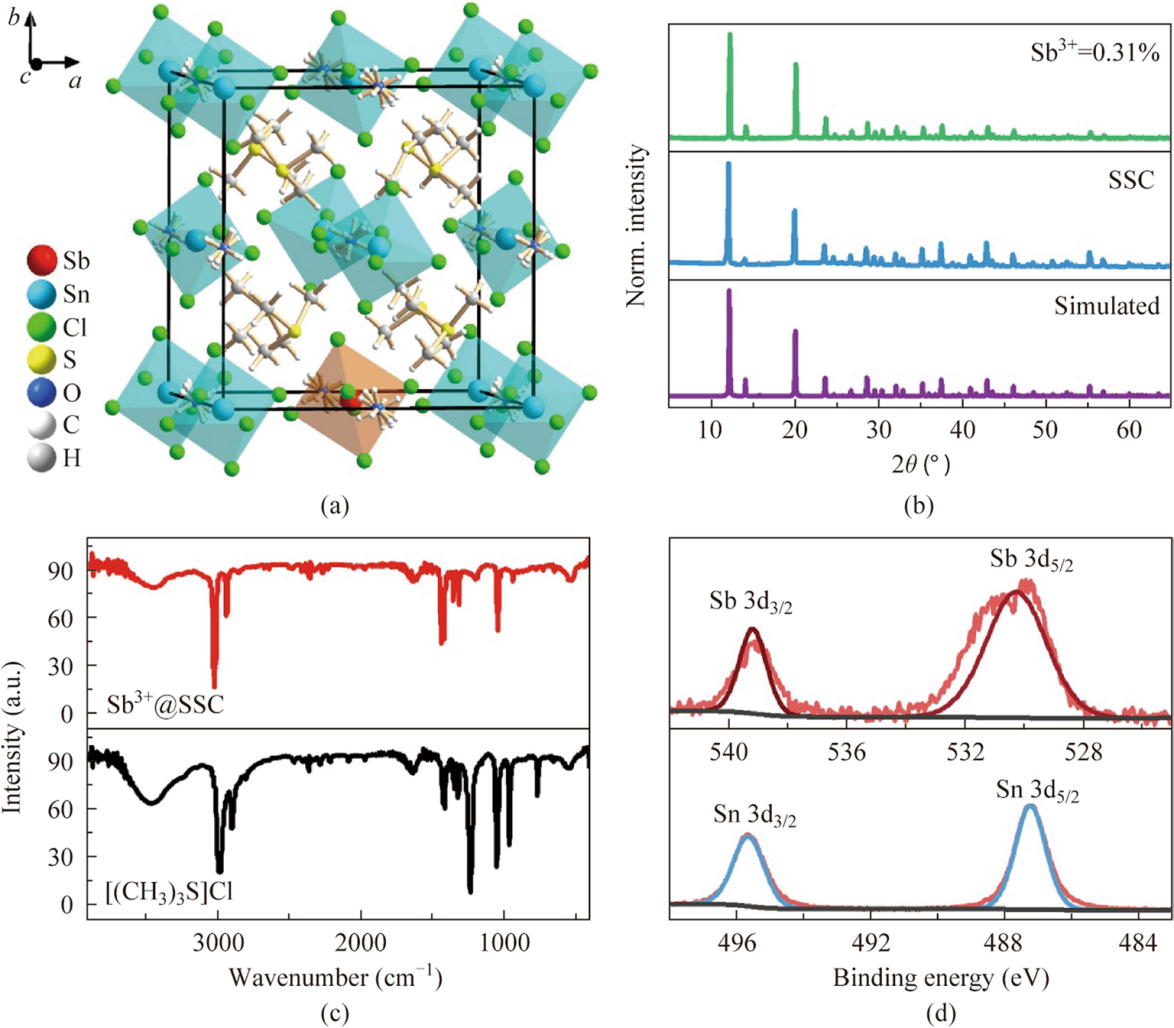
**a** Crystal structure of Sb^3+^@SSC. **b** Experimental and simulated powder X-ray diffraction (PXRD) of undoped host crystal and the experimental PXRD of 0.31% Sb^3+^@SSC. **c** Fourier ttransform infrared (FTIR) spectra of (CH_3_)_3_SCl and Sb^3+^@SSC. **d** X-ray photoelectron spectroscopy (XPS) spectra and peak fitting for Sn 3d and Sb 3d, respectively

SSC with/without dopant ions was synthesized by a hydrothermal method (The details can be found in Supporting Information). As shown in Fig. 1a, Sn is coordinated with six Cl forming an isolated [SnCl_6_]^2−^. The large organic cation [(CH_3_)_3_S]^+^ and H_2_O are located in the vacancy among the [SnCl_6_]^2−^, leading to a vacancy-ordered 0D MHPs SSC. The elemental analysis could confirm the existence of H_2_O as shown in Table S1. The crystal belongs to the *Pa*-3 space group with a unit cell length of 12.42 Å (Supplementary material 2). The Sb^3+^ dopant concentrations was tested by inductively coupled plasma atomic emission spectroscopy (ICP-OES), and the molar ratio of Sb/(Sn + Sb) was used to represent the actual dopant content inside the crystal structure. Under the feeding dopant concentrations of 0.002, 0.010, 0.018, 0.026, and 0.030 mol/L, the corresponding Sb^3+^ molar concentrations inside crystals was 0.019%, 0.12%, 0.25%, 0.31%, and 0.38%, respectively. As shown in Figs. 1b and S1, the good agreement between the experimental and simulated powder X-ray diffraction (PXRD) confirms the pure crystalline phase of title crystals. As shown in Fig. 1c, the Fourier transform infrared spectra (FTIR) of Sb^3+^@SSC is consistent with that of (CH_3_)_3_SCl verifying the organic component in the crystal structure. The characteristic peaks for C, S, Cl, Sn, and Sb elements could be found in the full X-ray photoelectron spectroscopy (XPS) spectrum of Sb^3+^@SSC as shown in Fig. S2. The fine XPS spectra of Sb and Sn in Sb^3+^@SSC are shown in Fig. 1d. The peaks located at 539.13 and 530.31 eV are in accordance with 3d_3/2_ and 3d_5/2_ of Sb^3+^, respectively, while those at 495.66 and 487.21 eV are contributed by 3d_3/2_ and 3d_5/2_ of Sn^4+^, respectively [24, 32, 38]. Thus, the elements and valence in Sb^3+^@SSC are well confirmed.

**Fig. 2:**
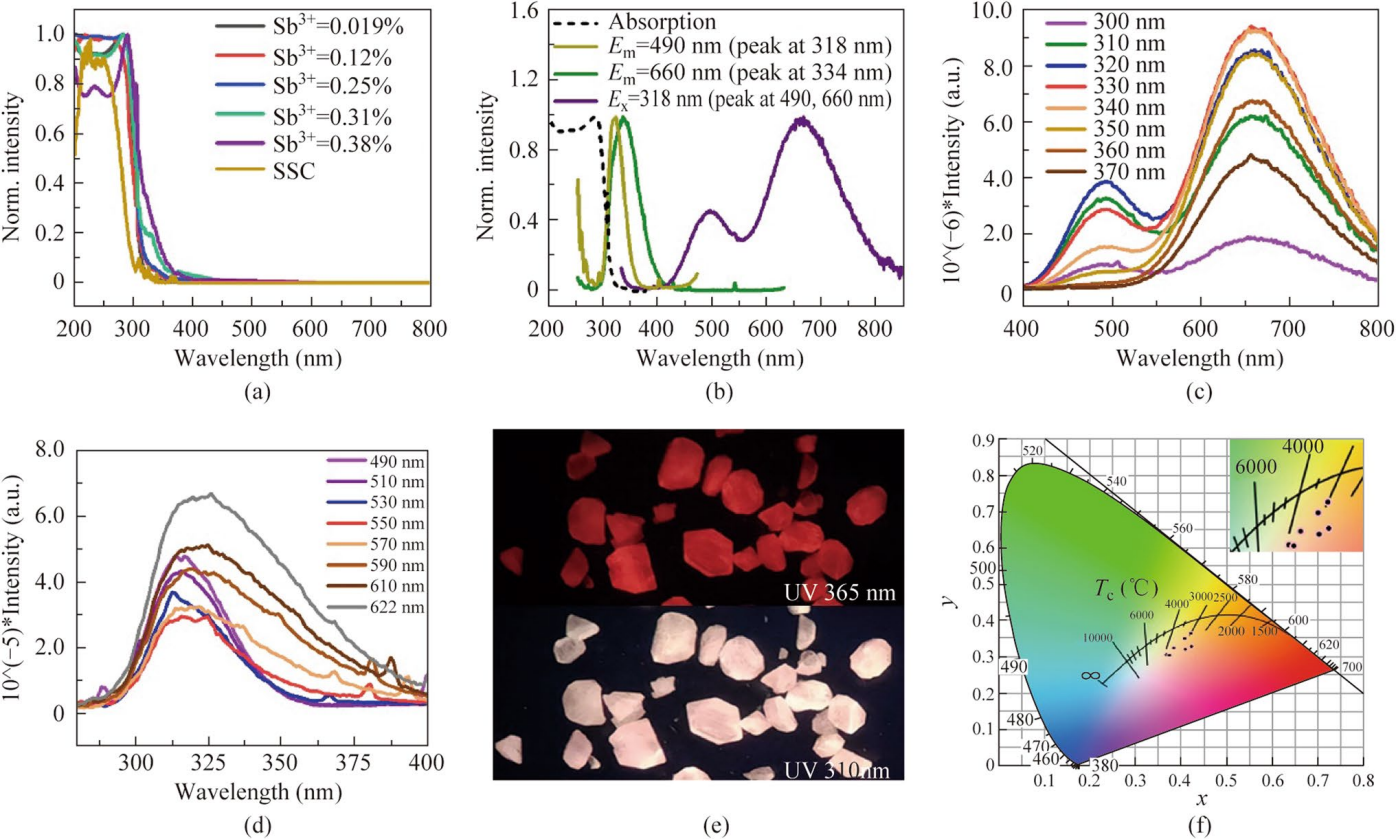
**a** UV–vis absorption spectra of *x*Sb^3+^@SSC (*x* = 0, 0.019%, 0.12%, 0.25%, 0.31%, and 0.38%). **b** UV–vis absorption (black line), photoluminescence excitation (PLE, yellow and green lines), photoluminescence emission (PL, purple line) spectra of 0.31% Sb^3+^@SSC. **c** PL spectra of 0.31% Sb^3+^@SSC under different excitation wavelengths. **d** PLE spectra of 0.31% Sb^3+^@SSC under different emission wavelengths. **e** Optical photographs of the single-doped samples under 365 and 310 nm light. **f** Commission Internationale de l´Eclairage (CIE) coordinates of 0.31% Sb^3+^@SSC under different excitation wavelengths

The optical properties of Sb^3+^@SSC are studied hereafter. Figure 2a is the UV–vis absorption spectra of *x*Sb^3+^@SSC (*x* = 0, 0.019%, 0.12%, 0.25%, 0.31%, and 0.38%). Figure 2b shows the UV–vis absorption (black line), photoluminescence excitation (PLE, yellow and green line), photoluminescence emission (PL, purple line) spectra of 0.31% Sb^3+^@SSC. The absorption peak around 290 nm arises from the host SSC [39]. Along with the increasing Sb^3+^ dopant, an additional peak around 340 nm emerges and rises due to the absorption of Sb^3+^ [18, 40]. This absorption peak induced by Sb^3+^ dopant can also be found in the PLE spectra as shown in Fig. 2b. The PL of pristine SSC is negligible. After Sb^3+^ doping, SSC can induce a broad emission with two peaks at 490 nm (named as S) and 660 nm (named as T) under 318 nm excitation light source. The optimal excitation wavelength at 490 nm is 318 nm (The yellow curve in Fig. 2b), while the optimal excitation wavelength at 660 nm is 334 nm (The green curve in Fig. 2b). As a result, Sb^3+^@SSC shows an excitation-dependent PL (Fig. 2c). When the excitation wavelength changes from 300 to 370 nm, the intensity ratio between S peak and T peak is principally decreased, exhibiting different PL color as shown in Figs. 2e and S3. The PLE spectra with emission wavelength from 490 to 622 nm show different shapes and features (Fig. 2d), suggesting that the broad emission composed of two PL peaks might originate from the relaxation of different excited states. As is the case for Sb^3+^ doped Cs_2_SnCl_6_ at 80 K, the emission peak at 490 nm is derived from ^1^P_1_ to ^1^S_0_, and the emission peak at 660 nm is derived from ^3^P_1_ to ^1^S_0_ [24]. The crystal emission close to the standard white light is obtained under the excitation light source of 340 nm (Commission Internationale de l´Eclairage, CIE = 0.37, 0.31) with color rendering index (CRI) 84. The color coordinates, correlated color temperature (CCT) and CRI of white light crystals obtained by different excitation light sources are shown in Table S2.

**Fig. 3:**
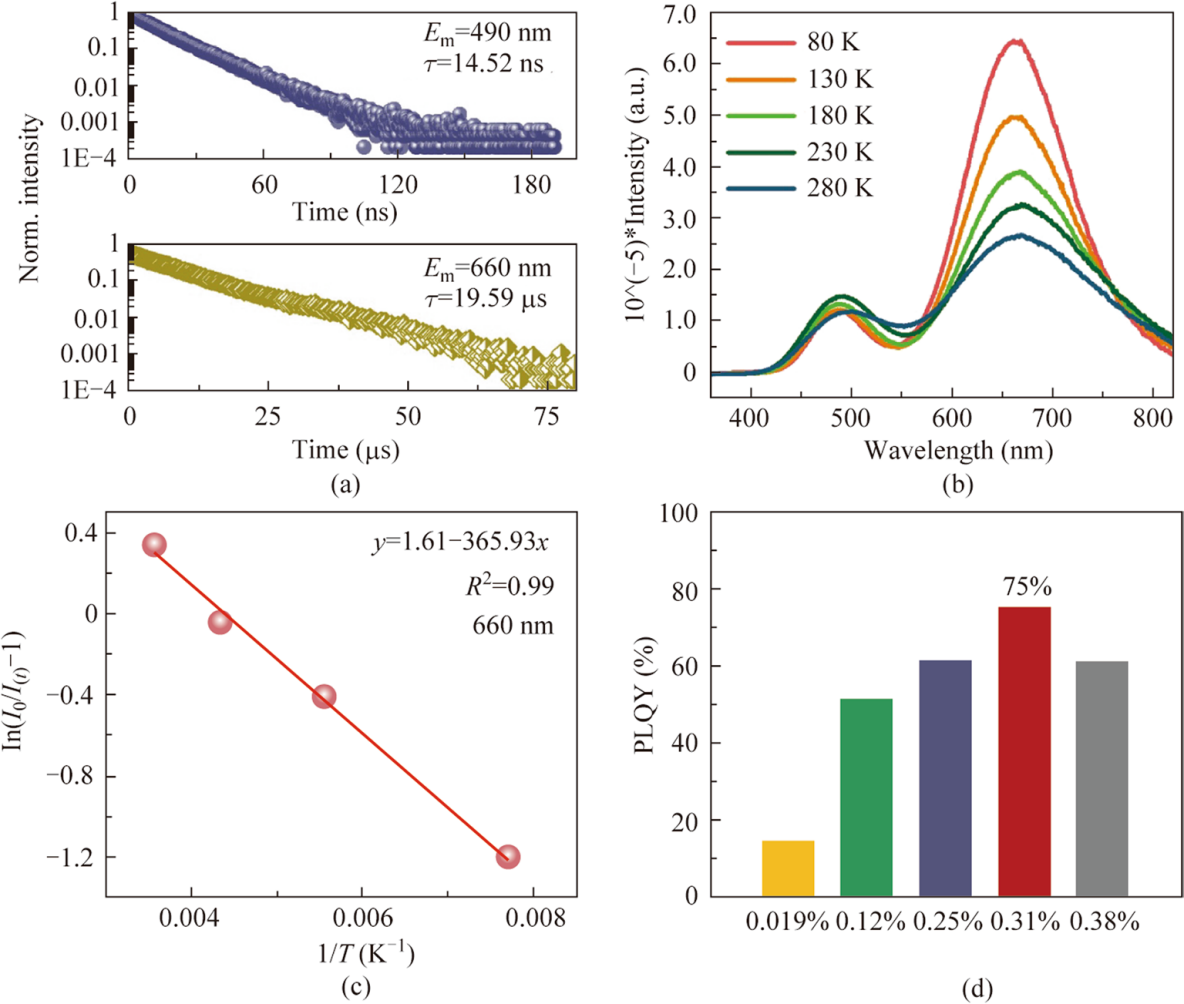
**a** Photoluminescence emission (PL) decay curves of 0.31% Sb^3+^@SSC at the peaks of 490 and 660 nm. **b** Temperature-dependent PL spectra under 327 nm excitation of 0.31% Sb^3+^@SSC. **c** T peak (i.e., 660 nm) intensity *I*_(*t*)_ under different temperatures for Sb^3+^@SSC. A deformed Arrhenius equation is fitted in the graph. **d** Photoluminescence quantum yield (PLQY) of *x*Sb^3+^@SSC for different values of *x*, at excitation of 330 nm

To understand the PL of Sb^3+^@SSC, the PL lifetimes for different peaks are measured as shown in Fig. 3a. The PL lifetime of the 490 nm peak is about 14.52 ns, while that of 660 nm peak is about 19.59 μs. The fitting parameters for PL lifetime are shown in Table S3. Thus, the singlet emission (^1^P_1_–^1^S_0_) and triplet emission (^3^P_1_–^1^S_0_) of Sb^3+^ in SSC crystal should give rise to the 490 and 660 nm peaks, respectively [41]. The temperature-dependent PL also takes place (Fig. 3b). When the temperature increases, the intensity of the 660 nm peak reduces rapidly, while the intensity of 490 nm peak enhances a little and then decreases. Two factors can be expected to affect the two peaks’ intensities at different temperatures. One is the electron–phonon coupling and the other is the thermal activated energy transfer between the singlet and triplet states. It seems that the electron–phonon coupling is dominant for the T peak, resulting in the decrease of the peak intensity. However, the energy transfer from the triplet state to singlet state (i.e., reverse intersystem crossing) might play a critical role for S peaks, leading to the increase of the peak intensity initially. The intensity of the T peak at different temperatures is fitted by Arrhenius equation (Eq. (1)), as shown in Fig. 3c.

1 $${I}_{(t)}=\frac{{I}_{0}}{1+a{\text{exp}}({E}_{{\text{A}}}/(KT))},$$

where *I*_0_ is the initial luminescence intensity emitted at 660 nm at low temperature (80 K), *K* is the Boltzmann constant, *a* is the pre-exponential factor, and *E*_A_ is the activation energy of the luminescence peak. The *E*_A_ of 660 nm emission calculated by the above formula is 265.17 meV; this value is similar to that in our group’s previous work on Sb^3+^ doped [(CH_3_)_3_N]_2_SnCl_6_ (288.13 meV) [38].

As shown in Fig. 3d, the PLQY of Sb^3+^ at different doping concentrations is tested. As further shown in Fig. S4, when the actual doping concentrations with Sb^3+^ are 0.019%, 0.12%, 0.25%, 0.31%, and 0.38%, the corresponding PLQY values are 15.5%, 51.8%, 61.5%, 75%, and 61.7%, respectively. When Sb^3+^ concentration ranges from 0.019% to 0.31%, the PLQY will be enhanced with the increasing Sb^3+^ content, reaching the maximum value of 75% at 0.31% dopant concentration. Subsequently, increase of Sb^3+^ doping content leads to the decrease of PLQY due to the concentration quenching effect [42].

**Fig. 4:**
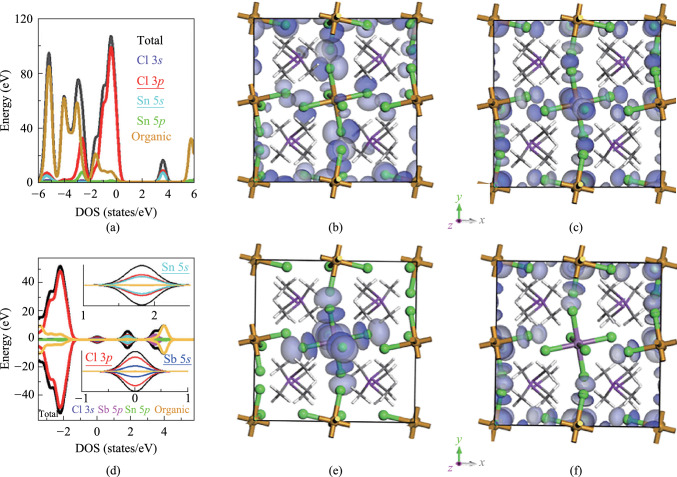
**a** Density of states of SSC. **b** and **c** represent the highest occupied molecular orbital (HOMO) and the lowest unoccupied molecular orbital (LUMO) of SSC, respectively. **d** Density of states of Sb^3+^@SSC. The insets represent the local enlarged figures near 0 and 2 eV. **e** and **f** represent the HOMO and LUMO of Sb^3+^@SSC, respectively

The density functional theory (DFT) calculation of SSC is shown in Fig. 4a–c. The highest occupied molecular orbital (HOMO) is mainly composed of Cl 3*p* with a small contribution from organic cation, while the lowest unoccupied molecular orbital (LUMO) is mainly composed of Cl 3*p* and Sn 5*s*. After Sb^3+^ doping, LUMO remains Cl 3*p* and Sn 5*s*, while Sb 5*s* and Cl 3*p* have obvious contribution to HOMO (Fig. 4d–f). The Cl atom exhibits spatial overlap on HOMO and LUMO in the Sb^3+^@SSC, which may result in a large energy separation between the lowest excited triplet state and the lowest excited singlet state [21, 43, 44]. The energy difference between singlet emission and triplet emission of Sb^3+^@SSC is 0.65 eV, comparable with that of Sb^3+^doped [(CH_3_)_4_N]_2_SnCl_6_, suggesting the large energy separation between the lowest excited triplet state and the lowest excited singlet state [38]. Thus, not all the electrons in the singlet state transfer into the triplet state, due to the small spin–orbit coupling (SOC). There are still some electrons undergoing radiative transition from the singlet energy level resulting in a spectrum with singlet–triplet dual emission peaks for Sb^3+^@SSC. The calculated bandgaps and experimental values of SSC and Sb^3+^@SSC are shown in Figs. S5 and S6. Due to the limitation of the generalized gradient approximation, the calculated bandgaps are smaller than the experimental values [45, 46].

**Fig. 5:**
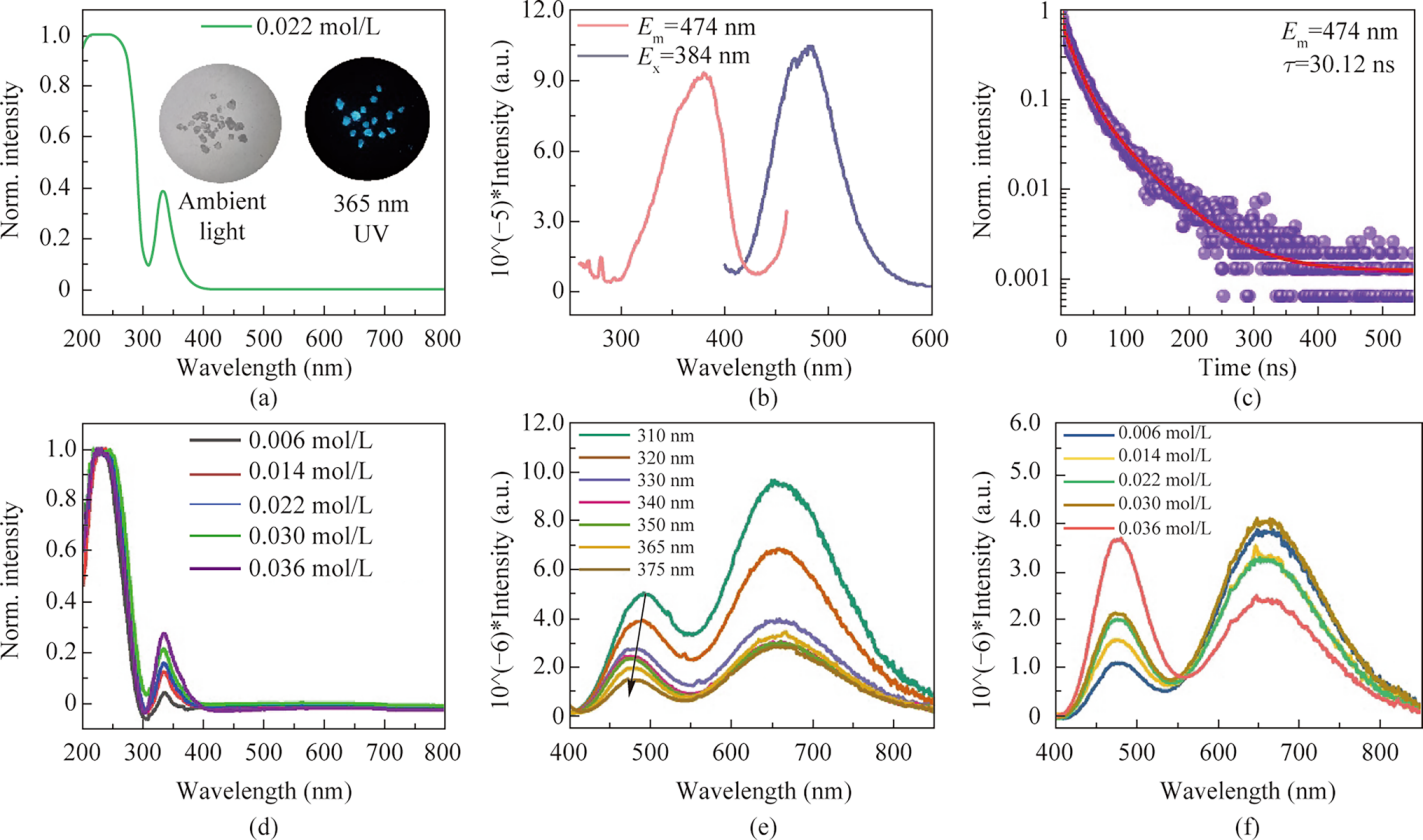
**a** UV–vis absorption spectrum of 0.022 mol/L Bi^3+^@SSC. The attached photos are Bi^3+^@SSC in ambient light and 365 nm light. **b** Photoluminescence excitation (PLE) and photoluminescence emission (PL) spectra of 0.022 mol/L Bi^3+^@SSC. **c** PL lifetime spectrum and fitting line of 0.022 mol/L Bi^3+^@SSC excited at 384 nm. **d** UV–vis absorption spectra of *x*Bi^3+^/0.31% Sb^3+^@SSC; the feeding concentrations are *x* = 0.006, 0.014, 0.022, 0.030, and 0.036 mol/L. **e** PL spectra of 0.022 mol/L Bi^3+^/0.31% Sb^3+^@SSC under different excitation wavelengths. **f** PL spectra of *x*Bi^3+^/0.31% Sb^3+^@SSC excitation at 365 nm; the feeding concentrations are *x* = 0.006, 0.014, 0.022, 0.030, and 0.036 mol/L

In addition to Sb^3+^ doping, Bi^3+^ is doped into SSC to obtain blue-emitting crystals. This is consistent with previous literatures about Bi^3+ ^doped Cs_2_HfCl_6_ and Bi^3+ ^doped Cs_2_ZrCl_6_ [47, 48]. The UV-vis absorption spectrum of Bi^3+^ doped [(CH_3_)_3_S]_2_SnCl_6_·H_2_O (Bi^3+^@SSC) is shown in Fig. 5a. After Bi^3+^ doping, an exciton peak appears at about 340 nm arising from the absorption of Bi^3+^ [40]. As shown in Fig. 5b and c, the blue emission peak (474 nm) of Bi^3+^@SSC is obtained under the 384 nm excitation with the PL lifetime of 30.12 ns. The blue light emission may be related to the mixing of the *sp* excited state of Bi^3+^ [40, 49].

As shown in Figs. S7 and S8, the introduction of Bi^3+^ does not change the structure of the SSC crystal. When Bi^3+^ is co-doped into Sb^3+^@SSC, the PL of Bi^3+^/Sb^3+^@SSC can be tuned efficiently by different Bi^3+^ concentrations. As shown in Fig. 5d, Bi^3+^/Sb^3+^@SSC has an obvious absorption peak at 340 nm, most of which is contributed by Bi^3+^. With the increase of Bi^3+^ ion concentration, the intensity of the absorption peak at 340 nm is increased. The emission peak of Bi^3+^@SSC is 474 nm with optimal excitation 384 nm and the S peak of Sb^3+^@SSC is at 490 nm with optimal excitation 318 nm. Thus, under different excitation wavelengths, ranging from 310 to 375 nm, as shown in Fig. 5e, the short-wavelength peak of co-doped sample is blue-shifted (i.e., from 490 to 474 nm), while the long-wavelength peaks (660 nm) are almost motionless. The doping with Bi^3+^ causes the white light emission of co-doped crystal to be excited at a longer wavelength (384 nm) compared with the 318 nm for Sb^3+^@SSC. The fluorescence emission spectra of *x*Bi^3+^/0.31% Sb^3+^@SSC (*x* is the feeding concentration for Bi^3+^, including 0.006, 0.014, 0.022, 0.030, and 0.036 mol/L) are shown in Fig. 5f. The fluorescence emission peak intensity at 474 nm is significantly enhanced with the increased Bi^3+^ concentration. The corresponding CIE, CCT, CRI and PLQY of the co-doped samples are listed in Table S4 and Fig. S9. Doping concentration of 0.006 mol/L Bi^3+^/0.31% Sb^3+^@SSC has the highest PLQY value of 29.6% under 330 nm excitation.

**Fig. 6:**
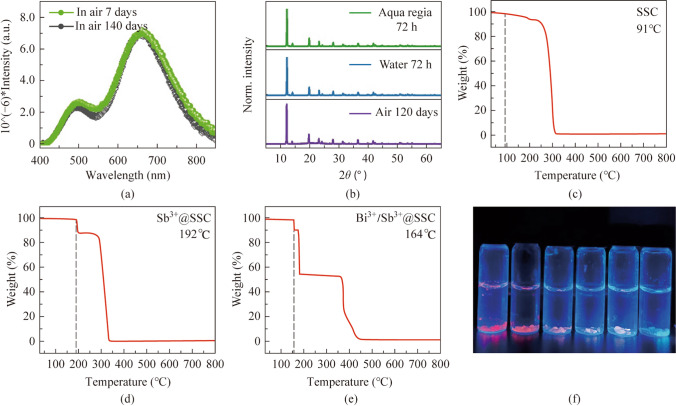
**a** Photoluminescence emission (PL) spectra of 0.31% Sb^3+^@SSC in air for 7 days and 140 days (under 331 nm excitation) to confirm the superior stability of Sb^3+^@SSC. **b** Powder X-ray diffraction (PXRD) of 0.31% Sb^3+^@SSC soaked in aqua regia, water, and air for 72 h, 72 h, and 120 days, respectively. The thermogravimetric (TG) curves of the **c** undoped, **d** single-doped, and **e** co-doped samples, respectively. **f** Optical photograph of single doped and co-doped samples in water (the left sample is Sb^3+^@SSC, and the middle sample is *x*Bi^3+^/0.31% Sb^3+^@SSC under 365 nm light. The feeding concentrations are *x* = 0.006, 0.022, 0.030, and 0.036 mol/L). The right one is 0.022 mol/L Bi^3+^@SSC)

Organic sulfonium cations have advantages of good humidity resistance [37, 50]. Figure 6a and b are the fluorescence stability tests of Sb^3+^@SSC in air. After 7 days and after 140 days in air, the fluorescence intensity does not change significantly. In addition, Sb^3+^@SSC immersed in water and aqua regia for 72 h. The PXRD after the experiment confirmed that Sb^3+^@SSC did not undergo phase transition in water or in aqua regia, as shown in Fig. 6b. The corresponding images for doped crystals soaked in water are shown in Fig. 6f. The samples can still maintain good luminescence properties after soaking in water. The thermal stability of single doped and co-doped samples is characterized by a thermogravimetric curve. As shown in Fig. 6c–e, the weight loss starts for pristine sample at 91 °C, while those for Sb^3+^ doped and Bi^3+^/Sb^3+^ co-doped samples it starts at 192 °C and 164 °C, respectively. The stability after doping is significantly improved compared with the undoped stability. The good stability provides a basis for the further application of such luminescent materials.

We assembled 0.31% Sb^3+^@SSC samples into LED devices. The electroluminescence spectrum of this device under 348 nm chip excitation is shown in Fig. S10. The emission spectrum covers a wide emission in the range of 380 to 760 nm. Under 300 mA current and 3 V voltage, the luminous efficiency is 4.39 lm/W. The mismatch between the ultraviolet LED chip and the optimal excitation wavelength leads to the low device efficiency.

## Conclusion

A new 0D SSC is synthesized. Bi^3+^ and Sb^3+^ are co-doped as dopants to tune the PL of the title crystals. As a result, Sb^3+^@SSC exhibits dual singlet/triplet emission at 490 and 660 nm, and the overall optical spectrum has a strong dependence on the excitation wavelength. Under excitation at 340 nm, the emission CIE (0.37, 0.31) is closest to the standard white light (CIE = 0.31, 0.31), and the CRI reaches 84 with 75% PLQY. The high energy barrier between singlet and triplet states might be the origin of dual emission in Sb^3+^@SSC at room temperature. The co-doping with Bi^3+^ increases the blue band emission in the white light. The white light color can be adjusted by changing the doping concentration ratio of Bi^3+^ and Sb^3+^. The excitation wavelength for white light can extend to 384 nm, which is convenient for WLED application. As a highly compatible host, SSC not only allows the doping with multiple ions, but also has enhanced stability, and provides a basis for further application.

## Supplementary Information

Below is the link to the electronic supplementary material.

Supplementary file1 (PDF 2016 KB)

Supplementary file2 (CIF 144 KB)

## Data Availability

The data that support the findings of this study are available from the corresponding author, upon reasonable request.
