# Correction: White light emission in 0D halide perovskite [(CH_3_)_3_S]_2_SnCl_6_·H_2_O crystals through variation of doping ns^2^ ions

**DOI:** 10.1007/s12200-024-00138-y

**Published:** 2024-11-12

**Authors:** Yitong Lin, Yu Zhong, Yangpeng Lin, Jiawei Lin, Lei Pang, Zhilong Zhang, Yi Zhao, Xiao-Ying Huang, Ke-Zhao Du

**Affiliations:** 1 Fujian Provincial Key Laboratory of Advanced Materials Oriented Chemical Engineering, Collage of Chemistry and Material Science, Fujian Normal University, Fuzhou, 350007 China; 2 State Key Laboratory of Structural Chemistry, Fujian Institute of Research on the Structure of Matter, Chinese Academy of Sciences, Fuzhou, 350002 China; 3 Qinghai Environmental Monitoring Center, Xining, 810000 China; 4 Strait Institute of Flexible Electronics (SIFE, Future Technologies), Fujian Normal University and Strait Laboratory of Flexible Electronics (SLoFE), Fuzhou, 350007 China; 5 Wuhan National Laboratory for Optoelectronics, Huazhong University of Science and Technology, Wuhan, 430074 China

**Correction: Frontiers of Optoelectronics (2024) 17:6** 10.1007/s12200-024-00109-3

Following publication of the original article [1], the authors reported the errors, which need to be corrected:

1. The sentence in Results and discussion section has been updated from “The crystal belongs to the *Fm*-3* m* space group with a unit cell length of 12.43 Å.” to “The crystal belongs to the *Pa-3* space group with a unit cell length of 12.42 Å.” A cif file has been added as a Supplementary file for comparison with other readers.
2. The sentence in Results and discussion section has been updated from “The energy difference between singlet emission and triplet emission of Sb^3+^ @SSC is 0.57 eV” to “The energy difference between singlet emission and triplet emission of Sb^3+^ @SSC is 0.65 eV”.
3. Fig. 5 has been updated from

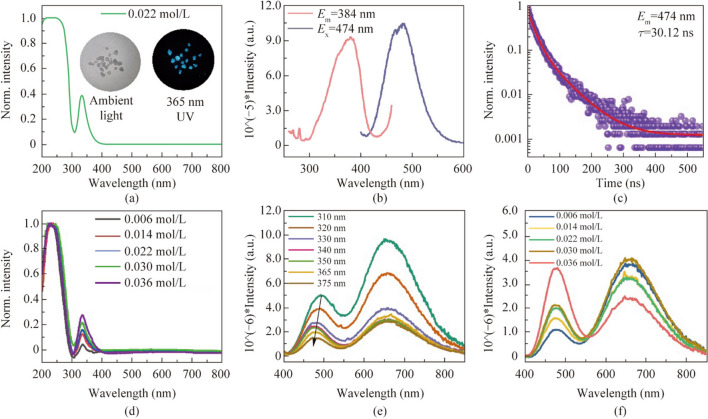


To:

**Fig. 5:**
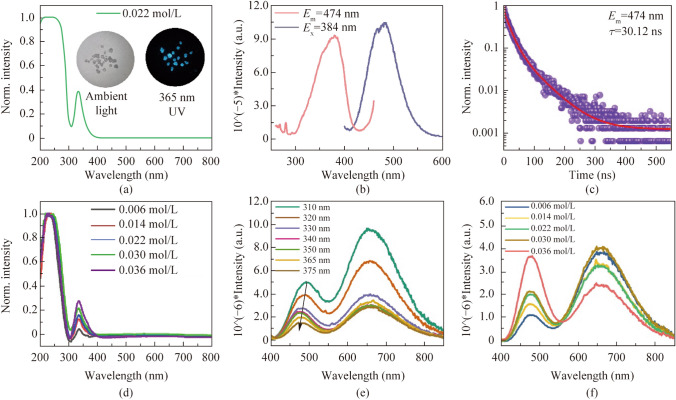
**a** UV–vis absorption spectrum of 0.022 mol/L Bi3 + @SSC. The attached photos are Bi3 + @SSC in ambient light and 365 nm light. **b** Photoluminescence excitation (PLE) and photoluminescence emission (PL) spectra of 0.022 mol/L Bi3 + @SSC. **c** PL lifetime spectrum and fitting line of 0.022 mol/L Bi3 + @SSC excited at 384 nm. **d** UV–vis absorption spectra of *x*Bi3 + /0.31% Sb3 + @SSC; the feeding concentrations are *x* = 0.006, 0.014, 0.022, 0.030, and 0.036 mol/L. **e** PL spectra of 0.022 mol/L Bi3 + /0.31% Sb3 + @SSC under different excitation wavelengths. **f** PL spectra of *x*Bi3 + /0.31% Sb3 + @SSC excitation at 365 nm; the feeding concentrations are *x* = 0.006, 0.014, 0.022, 0.030, and 0.036 mol/L.

The original article [1] has been updated.

## Supplementary Information

Supplementary file1 (CIF 144 KB)

## References

[1] Lin, Y., Zhong, Y., Lin, Y., Lin, J., Pang, L., Zhang, Z., Zhao, Y., Huang, X.Y., Du, K.Z.: White light emission in 0D halide perovskite [(CH3)3S]2SnCl6·H2O crystals through variation of doping ns2 ions. Front. Optoelectron. **17**, 6 (2024). 10.1007/s12200-024-00109-3 38374460 10.1007/s12200-024-00109-3 PMC10876505

