# Supplementary material for: White light emission in 0D halide perovskite [(CH3)3S]2SnCl6·H2O crystals through variation of doping ns2 ions
Source: Front Optoelectron. 2024 Feb 20;17(1):6. doi: 10.1007/s12200-024-00109-3 (PMC10876505; doi:10.1007/s12200-024-00109-3)
Supplement: Supplementary file 1 — Supplementary file1 (PDF 2016 KB) [file 12200_2024_109_MOESM1_ESM.docx]

**Supporting Information**

**White light emission in 0D halide perovskite [(CH_3_)_3_S]_2_SnCl_6_⋅H_2_O crystals through variation of doping ns^2^ ions**

Yitong Lin,^a^ Yu Zhong,^a,c,*^ Yangpeng Lin,^a^ Jiawei Lin,^a^ Lei Pang,^a^ Zhilong Zhang,^a^ Yi Zhao,^d,*^ Xiao-Ying Huang,^b^ Ke-Zhao Du^a,e*^

^a^ Fujian Provincial Key Laboratory of Advanced Materials Oriented Chemical Engineering, Collage of Chemistry and Material Science, Fujian Normal University, Fuzhou 350007, China

^b^ State Key Laboratory of Structural Chemistry, Fujian Institute of Research on the Structure of Matter, Chinese Academy of Sciences, Fuzhou 350002, China

^c^ Qinghai Environmental Monitoring Center, Xining 810000, China

^d^ Strait Institute of Flexible Electronics (SIFE, Future Technologies), Fujian Normal University and Strait Laboratory of Flexible Electronics (SLoFE), Fuzhou 350007, China

^e^ Wuhan National Laboratory for Optoelectronics, Huazhong University of Science and Technology, Wuhan 430074, China

*Corresponding authors: [duke@fjnu.edu.cn (Ke-Zhao Du)](mailto:duke@fjnu.edu.cn) [1007807055@qq.com (Yu Zhong)](mailto:1007807055@qq.com); [ifeyzhao@fjnu.edu.cn (Yi Zhao)](mailto:ifeyzhao@fjnu.edu.cn)

**Experimental Section**

**Materials and Chemicals:** Trimethyl Sulfoxide Chloride (C_3_H_9_SOCl, Adamas 99%), Diantimony Trioxide (Sb_2_O_3_ Adamas, 99%+), Tin (IV) Chloride Pentahydrate (SnCl_4_**⋅**5H_2_O, Adamas 99%+), Bismuth Trioxide (Bi_2_O_3_, Adamas, 99%+) and hydrochloric acid (HCl, 37~38% wt% in water, Sinopharm Chemical Reagent Co., Ltd., China, about 12 mol/L) were used without further purification.

**Preparation of dopant ion solution：** The commercial hydrochloric acid is diluted into 6 mol/L concentration by H_2_O. Firstly, 0.050 mol/L Sb^3+^ solution was obtained by dissolving 1.458 g (5 mmol) Sb_2_O_3_ in 100 ml diluted HCl. Then, 1 mL, 5 mL, 9 mL, 13 mL and 15 mL 0.050 mol/L Sb^3+^ solution was added into five vials, respectively. Finally, 6 mol/L HCl was used to dilute the Sb^3+^ solution into 0.002 mol/L, 0.010 mol/L, 0.018 mol/L, 0.026 mol/L and 0.030 mol/L, respectively.

0.050 mol/L Bi^3+^ solution was obtained by dissolving 2.330 g (5 mmol) Bi_2_O_3_ in 100 ml 6 mol/L HCl. Then, 3 mL, 7 mL, 11 mL, 15 mL and 18 mL 0.050 mol/L Bi^3+^ solution was added into five vials, respectively. Finally, 6 mol/L HCl was used to dilute the Bi^3+^ solution into 0.006 mol/L, 0.014 mol/L, 0.022 mol/L, 0.030 mol/L and 0.036 mol/L, respectively.

**Synthesis xSb^3+^@****SSC：**The stoichiometric amount of 76.8 mg (0.6 mmol) C_3_H_9_SOCl and 78.2 mg (0.3 mmol) SnCl_4_**⋅**5H_2_O were added in five polytetrafluoroethylene (PTFE)-lined stainless tanks with 3 ml different concentrations of Sb^3+^ acid solutions, respectively. The reactors were heated from room temperature to 130℃ within 300 min, and kept at this temperature for 1500 min. Then, the reactors were cooled to room temperature in 2000 min. Finally, the colorless crystals Sb^3+^@SSC with different Sb^3+^ concentrations can be collected. The crystals were washed by ethanol and dried in vacuum oven at 80°C for 1 h. The product yields were about 90%.

**Synthesis xBi^3+^/ 0.31% Sb^3+^@****SSC:** Firstly, the stoichiometric amount of 76.8 mg (0.6 mmol) C_3_H_9_SOCl and 78.2 mg (0.3 mmol) SnCl_4_**⋅**5H_2_O were added into five PTFE-lined stainless tanks. Secondly, 2.5 mL 0.026 mol/L Sb^3+^ solution was added into the five PTFE-lined stainless tanks. Thirdly, 2.5 mL Bi^3+^ solution with different concentrations was added into the five PTFE-lined stainless tanks, respectively. The other reaction conditions were the same as Sb^3+^@SSC. Finally, the title crystals were separated by filter, washed for three times with ethyl alcohol and dried in vacuum oven at 80°C for 1 h. The xBi^3^^+^/ 0.31%Sb^3+^@SSC colorless transparent crystal was obtained with the yield around 80%.

**Fabrication of LED Devices:** LED devices were constructed by coating the title compounds on the UV LED chips (310 nm and 365 nm). The title compounds were mixed with epoxy resin, respectively. Then, the obtained mixture was coated on the surface of the UV chips and solidified by heating at 80°C for 1 hour.

**Measurement and Characterization:** The single crystal diffraction test was performed on SuperNova produced by Rigaku (with Mo target, λ = 0.71073 Å). The crystalline phases of the obtained samples were determined by powder X-ray diffraction (PXRD) (Bruker D8 with Cu target, λ = 1.54178 Å). The Fourier Transform Infrared Spectrometer (FTIR) was analyzed by Thermo Scientific Nicolet 6700 FT-IR (400 ~ 4000cm^-1^, KBr tableting). XPS spectra were recorded on an AXIS Supra+ X-ray photoelectron spectrometer. Diffuse reflectance spectra were measured using Agilent CARY100 (The diffuse reflective layer on the inner wall of the integrating sphere is BaSO_4_, and the scanning range is 200 - 800 nm.). The photoluminescence (PL) and photoluminescence excitation (PLE) were recorded on a FLS1000 Spectrofluorometer - Edinburgh Instruments. The PL decay curves were measured on a FLS920-stm spectrometer-Edinburgh Instruments. The photoluminescence quantum yield (PLQY) was recorded on FS5 Spectrofluorometer-Edinburgh Instruments. The thermogravimetric analysis (TG) was performed on a NETZSCH STA-449F3 thermogravimetric analyzer in a dry atmosphere (N_2_) environment at a heating rate of 10 K/min. The concentration of the dopant ions in the title compounds were measured by Agilent ICP-OES730.

**Density functional theory (DFT) calculations**

The density functional theory (DFT) calculations were carried out by using the Materials Studio software interface through DMol^3^ module (BIOVIA, USA).[1, 2] The Perdew-Burke-Ernzerhof-General Gradient Approximation (PBE-GGA)[3] was used in all calculations. The double numerical polarization 3.5 as the basis set was employed in a global cutoff scheme of “fine”.[1] The quality and integration accuracy were all selected as “fine”. The atomic positions were optimized until the force on each atom was < 0.002 Ha/Å. The SCF tolerance was set as 10^-6^ eV. For the density of states and band structure of title compounds, their k-point mesh of 2 × 2 × 2 for the Brillouin zone sampling was employed. Then, for the charge distribution of title compounds, the Brillouin zone was sampled by using Gamma points. The other sets were the default.


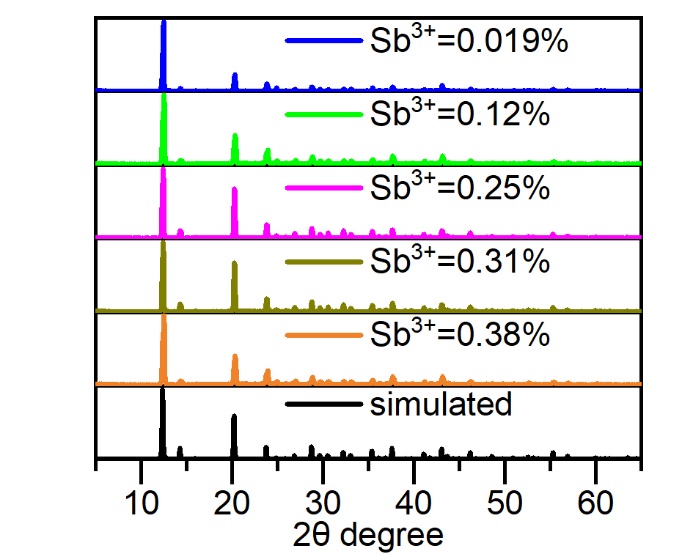


Fig. S1. The PXRD patterns of xSb^3+^@SSC (x = 0, 0.019%, 0.12%, 0.25%, 0.31% and 0.38%).


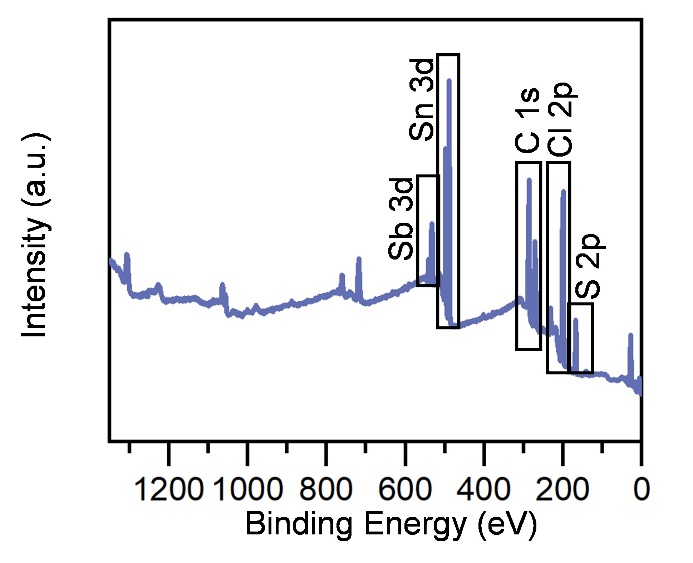


Fig. S2. The XPS survey spectrum of Sb^3+^@SSC.


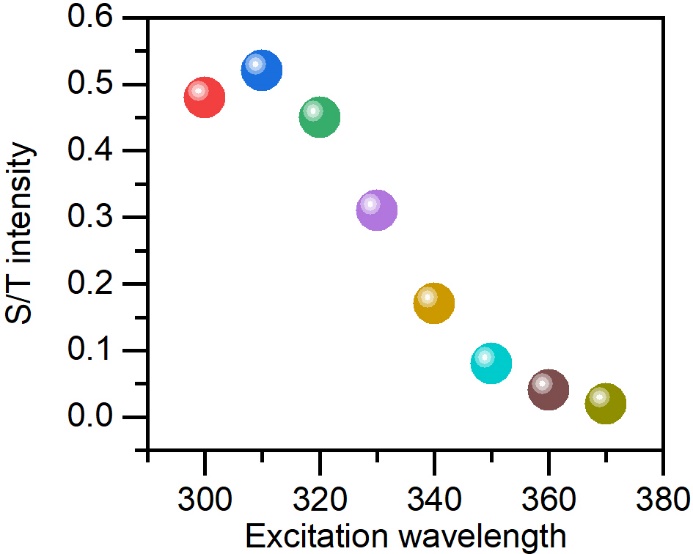


Fig. S3. Intensity ratio of 490 nm (named as S) and 660 nm (named as T) of 0.31% Sb^3+^@SSC under different excitation wavelengths.


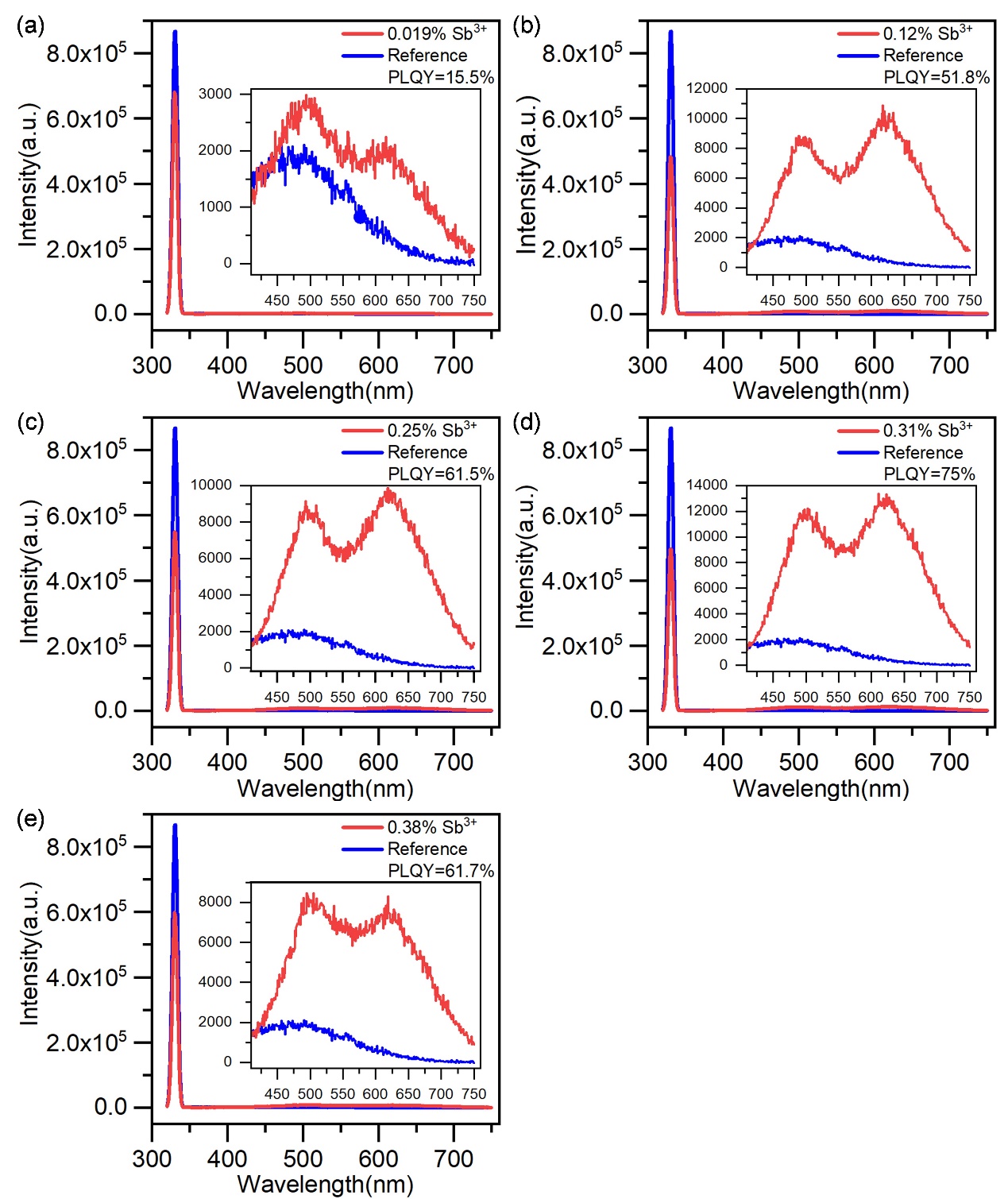


Fig. S4. The PLQY spectra of xSb^3+^@SSC (x = 0.019%, 0.12%, 0.25%, 0.31% and 0.38%).


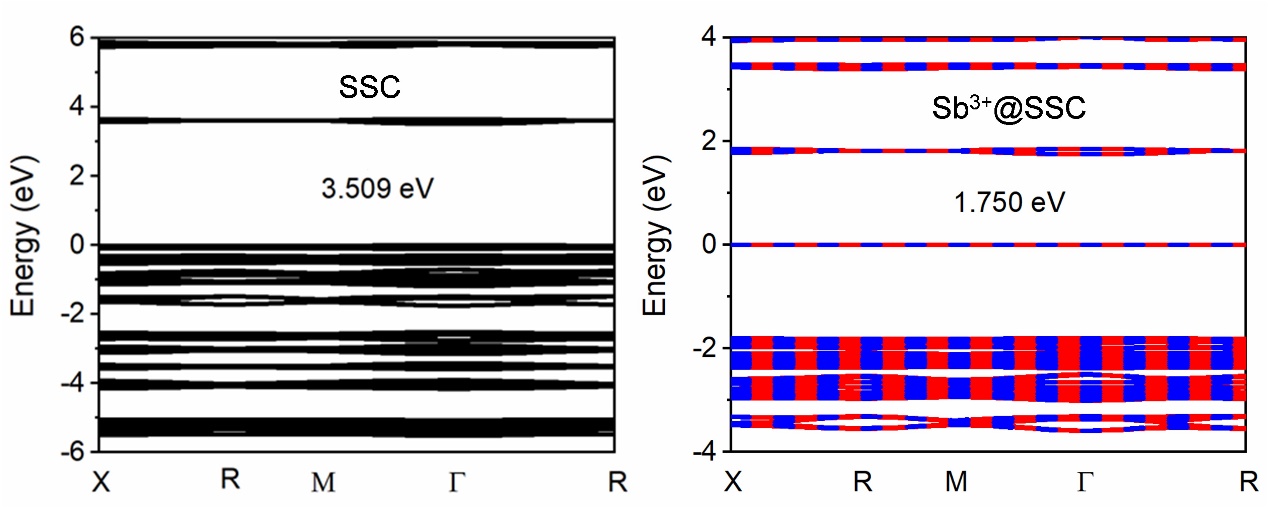


Fig. S5. The band structures of SSC and Sb^3+^@SSC.


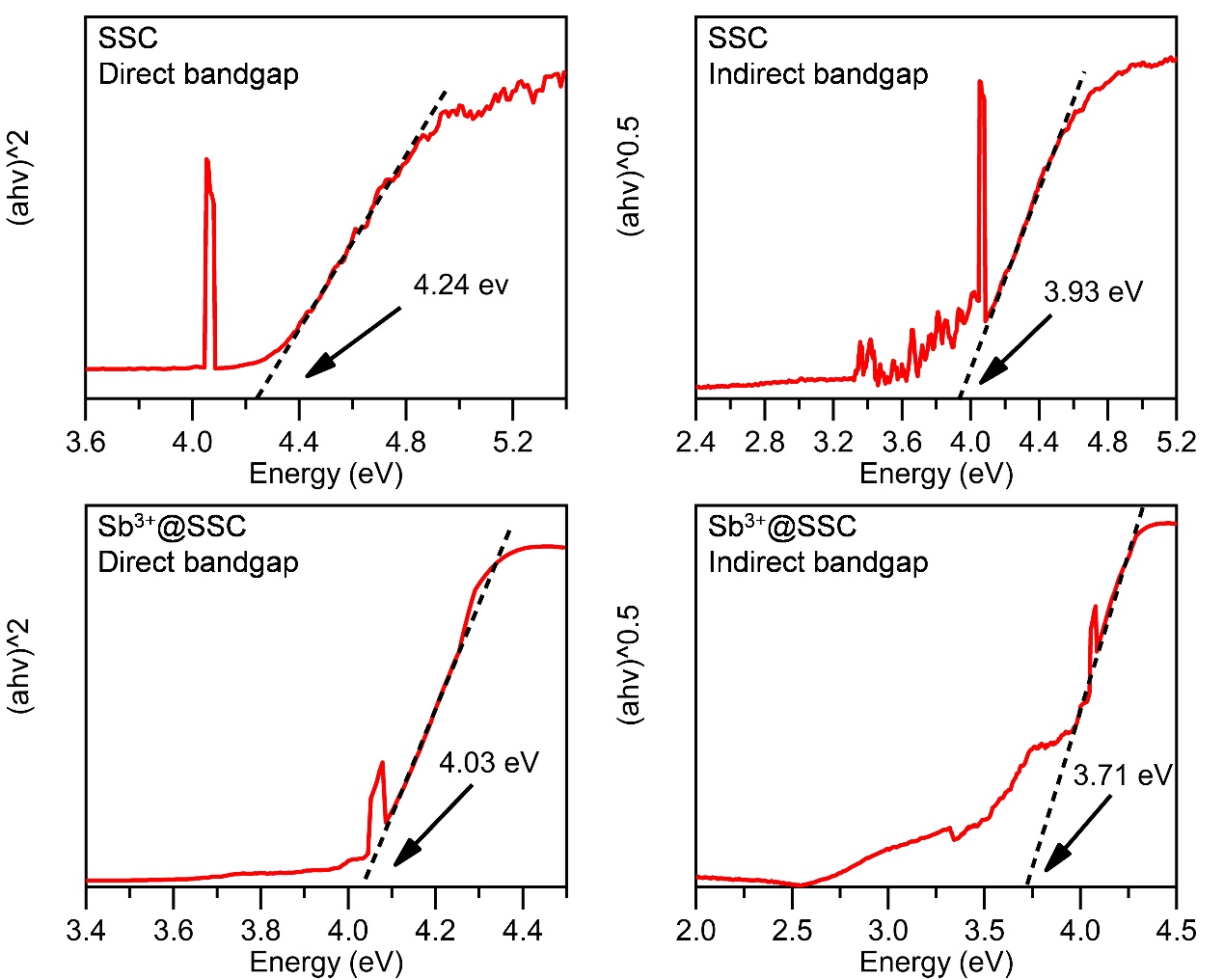


Fig. S6. The bandgap of SSC and Sb^3+^@SSC.


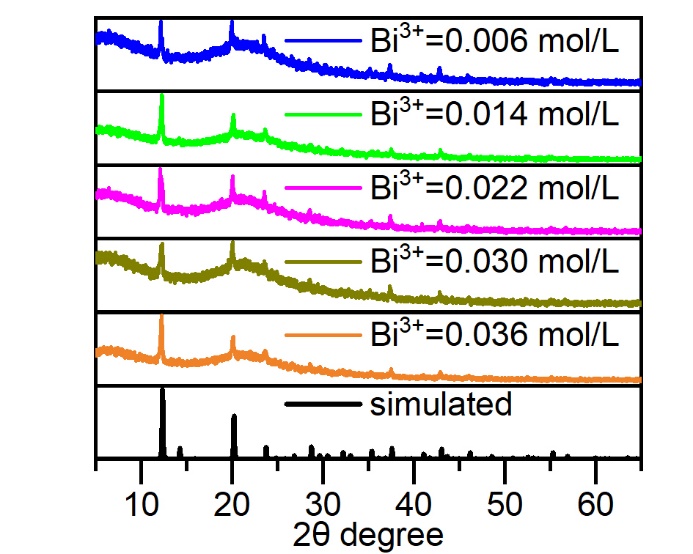


Fig. S7. The PXRD patterns of xBi^3+^@SSC (The feeding concentrations are x = 0, 0.006, 0.014, 0.022, 0.030 and 0.036 mol/L).


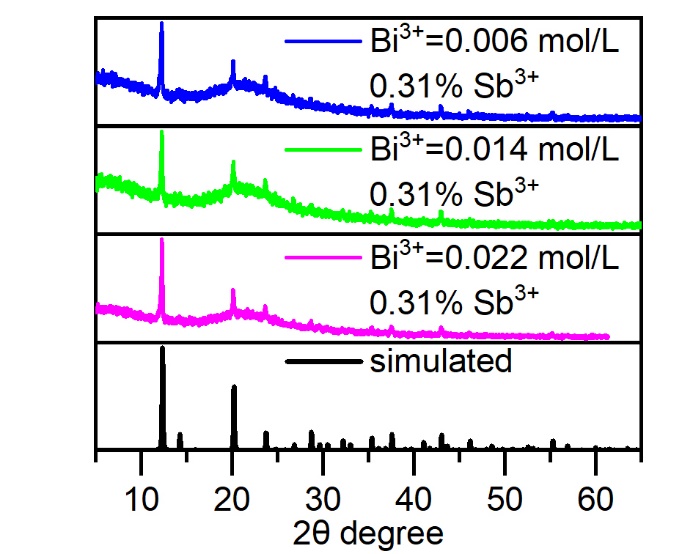


Fig. S8. The PXRD patterns of xBi^3+^/ 0.31% Sb^3+^@SSC (x = 0, 0.006, 0.014, 0.022 mol/L).


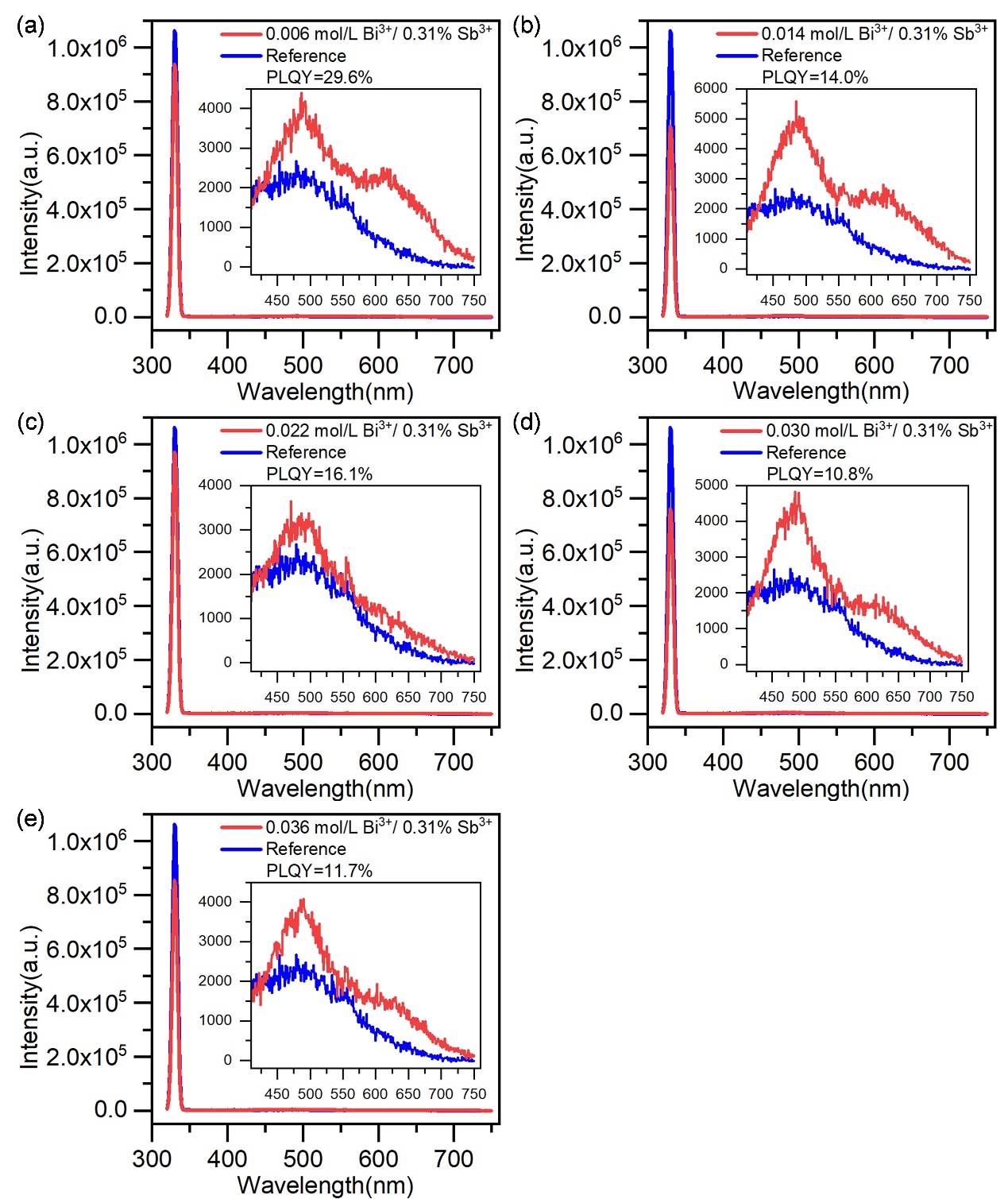


Fig. S9. The PLQY spectra of xBi^3+^/ 0.31% Sb^3+^@SSC (The feeding concentrations are x = 0.006, 0.014, 0.022, 0.030, 0.036 mol/L).


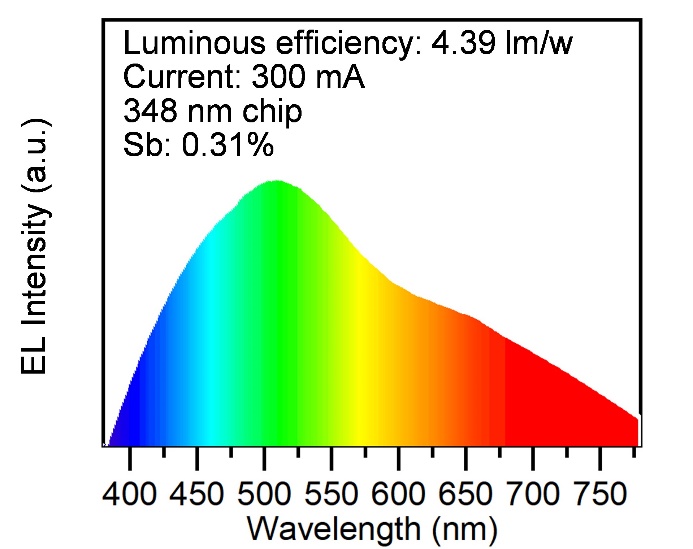


Fig. S10. The electroluminescent spectrum of 0.31% Sb^3+^@SSC. The excitation wavelength of LED chip is 348 nm.

Table S1. The elemental analysis of SSC.

| Elemental | Rate (%) |
| --- | --- |
| C | 14.83 |
| H | 3.41 |
| S | 13.68 |
| O | 5.964 |

Table S2. The CIE, CCT and CRI for the PL of 0.31% Sb^3+^@SSC under different excitation wavelength.

| Excitation wavelength | CIE | CCT | CRI |
| --- | --- | --- | --- |
| 310 nm | (0.42,0.37) | 2964 K | 66 |
| 320 nm | (0.41,0.35) | 3103 K | 70 |
| 330 nm | (0.38,0.33) | 3508 K | 78 |
| 340 nm | (0.37,0.31) | 3825 K | 84 |
| 350 nm | (0.37,0.30) | 3565 K | 79 |
| 365 nm | (0.41,0.32) | 2734 K | 60 |
| 375 nm | (0.43,0.33) | 2404 K | 50 |

Table S3. The decay curve fitting data of 0.31% Sb^3+^@SSC.

| Fitting date | 485 nm | 622 nm |
| --- | --- | --- |
| A_1_ | 0.48 ns | 0.99 μs |
| τ_1_ | 9.53 ns | 7.79 μs |
| A_2_ | 0.53 ns | 0.04 μs |
| τ_2_ | 17.05 ns | 51.30 μs |
| Τ_avg_ | 14.52 ns | 19.59 μs |
| R^2^ | 0.99 ns | 0.99 μs |

Table S4. CIE, CCT, CRI and PLQY of xBi^3+^/ 0.31% Sb^3+^@SSC under 330 nm excitation.

| Bi^3+^ feeding concentration | CIE | CCT | CRI | PLQY |
| --- | --- | --- | --- | --- |
| 0.006 mol/L | (0.47,0.34) | 1944 K | 28 | 29.6% |
| 0.014 mol/L | (0.42,0.32) | 2326 K | 47 | 14.0% |
| 0.022 mol/L | (0.40,0.32) | 2881 K | 64 | 16.1% |
| 0.030 mol/L | (0.38,0.32) | 3492 K | 78 | 10.8% |
| 0.036 mol/L | (0.30,0.28) | 7962 K | 79 | 11.7% |

**References**

1. Delley, B.: An all-electron numerical method for solving the local density functional for polyatomic molecules. J. Chem. Phys. 92, 508-517 (1990)

2. Delley, B.: From molecules to solids with the DMol^3^ approach. J. Chem. Phys. 113, 7756-7764 (2000)

3. Perdew, J.P., Burke, K. and Ernzerhof, M.: Generalized Gradient Approximation Made Simple. Phys. Rev. Lett. 77, 3865-3868 (1996)
